# A292 IMPACT OF SIROLIMUS PROTEINURIA FOLLOWING LIVER TRANSPLANTATION

**DOI:** 10.1093/jcag/gwad061.292

**Published:** 2024-02-14

**Authors:** R Kaviani, M Kruger, M Ma, J Abraldes, R Bhanji

**Affiliations:** Gastroenterology, University of Alberta, Edmonton, AB, Canada; Gastroenterology, University of Alberta, Edmonton, AB, Canada; Gastroenterology, University of Alberta, Edmonton, AB, Canada; Gastroenterology, University of Alberta, Edmonton, AB, Canada; Gastroenterology, University of Alberta, Edmonton, AB, Canada

## Abstract

**Background:**

Sirolimus (Sr) is a potent immunosuppressant used in liver transplant recipients to prevent rejection in settings of calcineurin inhibitor toxicity and in transplanted hepatocellular carcinoma (HCC) patients. Sr can cause proteinuria, which can lead to poor renal function and survival. The significance of proteinuria is poorly understood, with a lack of studies assessing its risk factors and their impact on clinical outcomes.

**Aims:**

We evaluated the incidence of proteinuria and its impact on clinical outcomes among liver transplant (LT) recipients who were Sr users compared with non-sirolimus (nonSr) users.

**Methods:**

We analyzed patients with their first LT between 2001 and 2020. Data were gathered from Organ Transplant Tracking records and chart reviews. Sr users received Sr for at least 6 consecutive months in the first year post-LT. We studied demographics, pre-LT comorbidities, and immunosuppression use. We evaluated the development of proteinuria, renal dysfunction, and new comorbidities including features of metabolic syndrome and cardiovascular disease. Data on post-LT infections, graft rejection, and patient survival were collected.

**Results:**

We analyzed 359 Sr and 762 non-Sr users (73.5% vs. 65% male). The average ages of Sr and non-Sr users were 54.9±9 and 51.7±11.6 years, respectively (pampersand:003C0.001). Of Sr users, 40.4% had HCC (pampersand:003C0.001). Among non-Sr users, 95% were on tacrolimus and 67.8% were on mycophenolate. Higher frequency of pre-LT hypertension (HTN) was observed in Sr users (27.2% vs. 18.2%; pampersand:003C0.001). There were no differences in pre-LT chronic kidney disease (CKD), cardiovascular disease (CVD), dyslipidemia (DLD), diabetes mellitus (DM) prevalence, creatinine levels, or proteinuria. Sr users had a higher incidence of proteinuria (13.1% vs. 7.9%, p=0.006). Protein-creatinine ratios and albumin-creatinine ratios 12 months post-LT were not significantly different in Sr and non-Sr users ([40.1±105.6 vs. 57.5±169.9 mg/g, p=0.39], and [56.2±101.4 vs. 54.6±166.8 mg/g, p=0.268], respectively). Pre-LT creatinine (OR1.5; pampersand:003C 0.001) and DM (OR2.7; pampersand:003C 0.001) were associated with an increased risk of proteinuria. Sr users had higher (pampersand:003C0.001) incidence of post-LT CVD (24% vs. 14.8%), DM (20.4% vs. 12.6%), HTN (41.5% vs. 30.3%), and DLD (41.3% vs. 20.3%). There was no difference in post-LT CKD between Sr vs non-Sr (41% vs. 47%; p =0.119). Sr users had a lower frequency of post-LT infection (43.1% vs. 53%, p=0.007). Higher incidence of graft rejection was seen in Sr users (43.5% vs 27.8%, pampersand:003C0.001). There was no difference in median graft (15.2 ± 0.40 vs. 14.4±0.30 years; p = 0.681) or patient survival (12.5±0.44 vs. 12.5±0.33 years; p = 0.536).

**Conclusions:**

Although Sr users were less likely to develop CKD post-LT, they had significantly higher rates of proteinuria, CVD, HTN, and DLD. However, no significant difference was observed in graft or patient survival rates.

**Funding Agencies:**

None

